# Two-dimensional optical spatial differentiation and high-contrast imaging

**DOI:** 10.1093/nsr/nwaa176

**Published:** 2020-08-06

**Authors:** Junxiao Zhou, Haoliang Qian, Junxiang Zhao, Min Tang, Qianyi Wu, Ming Lei, Hailu Luo, Shuangchun Wen, Shaochen Chen, Zhaowei Liu

**Affiliations:** Key Laboratory for Micro-/Nano-Optoelectronic Devices of Ministry of Education, School of Physics and Electronics, Hunan University, Changsha 410082, China; Department of Electrical and Computer Engineering, University of California, San Diego, La Jolla, CA 92093, USA; Interdisciplinary Center for Quantum Information, State Key Laboratory of Modern Optical Instrumentation, ZJU-Hangzhou Global Science and Technology Innovation Center, Zhejiang University, Hangzhou 310027, China; Department of Electrical and Computer Engineering, University of California, San Diego, La Jolla, CA 92093, USA; Department of Nano Engineering, University of California, San Diego, La Jolla, CA 92093, USA; Department of Electrical and Computer Engineering, University of California, San Diego, La Jolla, CA 92093, USA; Department of Electrical and Computer Engineering, University of California, San Diego, La Jolla, CA 92093, USA; Key Laboratory for Micro-/Nano-Optoelectronic Devices of Ministry of Education, School of Physics and Electronics, Hunan University, Changsha 410082, China; Key Laboratory for Micro-/Nano-Optoelectronic Devices of Ministry of Education, School of Physics and Electronics, Hunan University, Changsha 410082, China; Department of Nano Engineering, University of California, San Diego, La Jolla, CA 92093, USA; Department of Electrical and Computer Engineering, University of California, San Diego, La Jolla, CA 92093, USA

**Keywords:** metasurface, edge detection, spatial differentiation

## Abstract

Optical analog signal processing technology has been widely studied and applied in a variety of science and engineering fields, with the advantages of overcoming the low-speed and high-power consumption associated with its digital counterparts. Much attention has been given to emerging metasurface technology in the field of optical imaging and processing systems. Here, we demonstrate, for the first time, broadband two-dimensional spatial differentiation and high-contrast edge imaging based on a dielectric metasurface across the whole visible spectrum. This edge detection method works for both intensity and phase objects simply by inserting the metasurface into a commercial optical microscope. This highly efficient metasurface performing a basic optical differentiation operation opens up new opportunities in applications of fast, compactible and power-efficient ultrathin devices for data processing and biological imaging.

## Teaser

High-contrast broadband two-dimensional edge detection is demonstrated for both the intensity and phase objects, which brings fast and power-efficient metasurfaces for optical information processing and imaging one step closer to the reality.

## INTRODUCTION

As image processing becomes vital in various areas of science and technology, there is increasing demand for faster, integrated, efficient devices that can process optical signals and images. There are two common approaches for image processing: it is either conducted in the digital domain via integrated circuits or in an analog way based on optical components [1–3]. Although the digital method provides great versatility, it suffers from low operation speed and high energy consumption, leading to insurmountable challenges in the current big-data era. Therefore, optics-based analog signal processing methods have gained significant attention in recent years.

Within the last decade, the metasurface, a kind of planar optical element, has been used to manipulate light by employing the principle of diffraction [4,5]. Various optical devices have been developed including lenses [6–8], waveplates [9], holograms [10,11], polarizers [12,13] and spectral filters [14], of much lower weight and more advanced manipulation. Metasurfaces have evolved from the early plasmonic meta-atoms [15] to high refractive index dielectric materials [5] for improved energy efficiency and broadband capabilities. In recent years, interest has focused on practical applications of the metasurface, such as high resolution imaging [16], equation solver [17], analog optical computing [18,19] and, especially, spatial differentiators to achieve optical edge detection [20,21]. In addition, the high-cost fabrication methods such as e-beam lithography and focused ion beam lithography are being replaced by more cost-effective methods such as nano-imprint [22,23] and laser-writing methods [24].

Engineered nanophotonic materials have been widely studied for optical analog image processing, especially for edge detection, which has seen significant applications in machine and computer vision [25], medical imaging operation [26,27] and autonomous vehicles [28,29]. Recently, several theoretical works have investigated how to achieve spatial differentiation using optics [30–32]. Their methods typically rely on performing mathematical operations with designed metamaterials, which require complex material and fabrication processes. Furthermore, spatial differentiation has been extended to experimental demonstration by exploiting various approaches, including surface plasmonics [33,34], photonic crystals [35], the photonic spin Hall effect [36,37] and the Pancharatnam-Berry phase metasurface [38]. However, all of these current methods have their own limitations, and experimental demonstration of a highly efficient, compact, two-dimensional (2D) spatial differentiation device working for broadband frequencies is still lacking.

In the following, we propose a new design to impart 2D spatial differentiation on the impinging wavefront, based on the dielectric metasurface in transmission mode, providing advantages of high efficiency, broadband and high-contrast. The designed metasurface comprises a symmetric phase gradient along the radial direction, which enables linearly polarized (LP) beam splitting to left-handed circular polarization (LCP) and right-handed circular polarization (RCP) components along the radial direction and guarantees 2D spatial differentiation (see Supplementary data, Note 1). The dielectric metasurface based on a geometric phase without any resonance structure ensures operation at broadband working wavelength (whole visible range), enabling differentiation of color images. The high transmission mode of the compact metasurface sample facilitates alignment or integration with the rest of the optical system, which is important for imaging-processing applications. Our work provides new opportunities in optical analog computing and high-contrast imaging.

**Figure 1.**
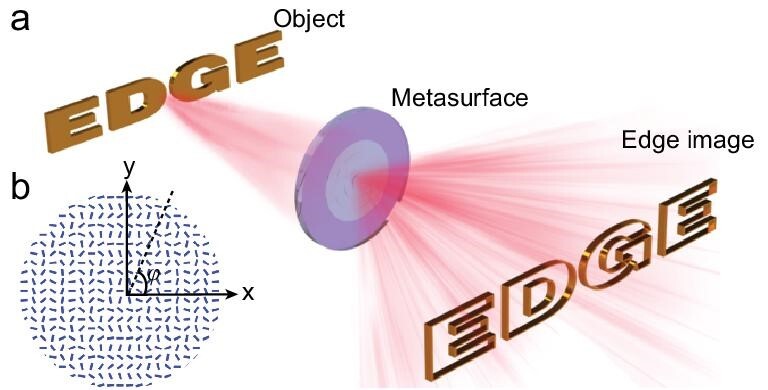
Schematic figure of the 2D edge detection. (a) The light incidents onto the ‘EDGE’ shaped object, then passes through the metasurface at the Fourier plane, and finally its edge information is obtained at the image plane. (b) Schematic figure of optical slow axis distribution of the mentioned metasurface.

To derive the edge detection formula, we take a one-dimensional (1D) case as an example to show the details. According to ref. [38], when the metasurface sandwiched between two orthogonal polarizers is placed on the Fourier plane of a 4*f* system, the amplitude of the output electric field of the object ${E_{\textit{in}}}( {{x_0},{y_0}} )$ can be given as $E( {x,y} )= {E_{\textit{in}}}( {{x_0} + {\rm{\Delta }},{y_0}} ) - {E_{\textit{in}}}( {{x_0} - {\rm{\Delta }},{y_0}} )$. Here, ${\rm{\Delta }} = \frac{{\lambda f}}{\Lambda }$, $\lambda $ is the working wavelength, *f* is the focal distance and $\Lambda $ is the period of metasurface. In case the shift ${\rm{\Delta }}$ is much smaller than the image feature size, for an intensity object ${E_{\textit{in}}}( {{x_0},{y_0}} ) = A( {{x_0},{y_0}} )$, the output electrical field distribution can be written as ${E_{out}}( {x,y} ) = A( {{x_0} + {\rm{\Delta }},{y_0}} ) - A( {{x_0} - {\rm{\Delta }},{y_0}} ) = 2{\rm{\Delta }}\frac{{\partial A}}{{\partial x}}$. For our current 2D edge detection, it can be regarded as superposition of infinite 1D edge detection processes that radially span the entire 2π azimuth angles in polar coordinates. So, using similar manipulation to the 1D case, the output electrical field for the 2D case under polar coordinates can be given as ${E_{out{\_}edge}}( {{\rm{r}},{\rm{\theta }}} ) \simeq 2{\rm{\Delta }}\frac{{\partial {\rm{A}}}}{{\partial {\rm{r}}}}$ for an intensity object. While for a phase object, ${E_{\textit{in}}}( {{x_0},{y_0}} ) = \exp [i{\rm{\Phi }}( {{x_0},{y_0}} )]$, the first order Taylor expansion is employed. The object ${E_{\textit{in}}}( {{x_0},{y_0}} )$ can be rewritten as $O( {{x_0},{y_0}} ) \approx 1 + {\rm{i\Phi }}( {{x_0},{y_0}} )$. The final electric field will be ${E_3}( {x,y} ) = i{\rm{\Phi }}( {{x_0} + {\rm{\Delta }},{y_0}} ) - i{\rm{\Phi }}( {{x_0} - {\rm{\Delta }},{y_0}} )$, which can be further expressed as ${E_{out{\_}edge}}( {x,y} ) \simeq 2{\rm{\Delta }}\frac{{\partial {\rm{\Phi }}}}{{\partial {\rm{r}}}}$. As shown schematically in Fig. 1(a), our designed system has the ability to filter the edge information of one object. Figure 1(b) schematically represents the optical axis distribution of the designed metasurface, in which the phase gradient is along both the *x* and *y* directions.

## EXPERIMENTAL RESULTS

### Sample information

**Figure 2.**
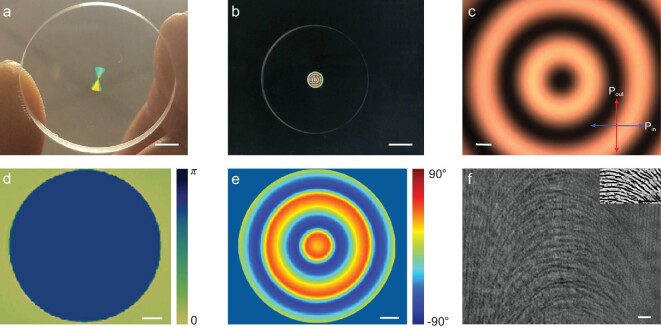
Characterization of the metasurface embedded in silica glass. (a) Photograph of a metasurface sample. Scale bar: 4 mm. (b) Polariscope image of the metasurface. Scale bar: 4 mm. (c) Zoomed polariscope optical image of the sample pattern area marked in (b). Scale bar: 200 μm. (d) The measured uniform phase retardance of the metasurface sample. Scale bar: 500 μm. (e) The pseudo color presents direction of the slow axis. Scale bar: 500 μm. (f) Polariscope optical image shows the finer structure of the metasurface. White scale bar: 3 μm. Inset, top view of scanning electron microscope (SEM). Black scale bar: 1 μm.

Figure 2(a) shows a photograph of the metasurface sample with patterned area (4 mm in diameter) in the center of a 1-inch SiO_2_ substrate. The thickness of the substrate is 3 mm. Figure 2(b) is a polariscope image of the sample, which reflects the form-birefringent characteristics of the metasurface area. The metasurface pattern was fabricated by scanning a femtosecond pulse laser inside the silica slabs (50 μm beneath the surface). The self-assembled nanostructures in silica slabs were formed under intense laser irradiation. By varying the laser polarization gradually, nanostructures with gradually changing orientation could be generated. More sample fabrication details can be found in previous works [39,40]. A zoomed polariscope optical image of the marked sample pattern area of Fig. 2(b) is illustrated in Fig. 2(c). Figure 2(d) shows the measured constant value of phase retardance of the metasurface sample, which works as a half-wave plate and ensures the conversion efficiency of the incident LP beam to RCP and LCP components [41]. For our metasurface, the measured conversion efficiency is close to unity at the working wavelength. The corresponding transmission efficiency (the ratio between the transmitted power and the incident power) reaches as high as 95%, which is higher than the achromatic metalenses with ∼50% transmission efficiency demonstrated in refs [42,43]. Figure 2(e) indicates the measured slow axis characterization inside the silica glass, which is along the radial direction of the birefringent sample. The orientation of the slow axis $\varphi ( {x,y} )$ ranges from 0 to π. As a result, the phase profiles of geometric phase elements (metasurface) experience a relative phase change, which is equal to $2\varphi ( {x,y} )$, i.e. from 0 to 2π [44]. More measurement details related to phase retardance and slow axis characterization can be found in the Methods. Figure 2(f) shows the finer structure of the metasurface with polariscope image along the radial direction.

### Transfer function demonstration

**Figure 3.**
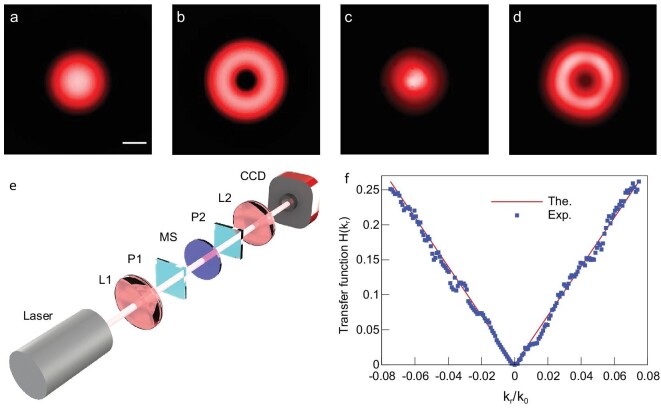
Measurement of the spatial transfer function of the metasurface spatial differentiator. (a and b) The calculated result without and with the spatial differentiator, respectively. Scale bar: 500 μm. (c and d) The corresponding experimental results. (e) Experiment setup: L, lens, focal length 25 mm; P1 and P2, a pair of crossed polarizers; MS, metasurface, period 1000 μm; CCD, charge couple device. (f) Theoretical and experimental results of the transfer function.

To demonstrate the spatial differentiation function, the transfer function of the metasurface sample was measured. As shown in Fig. 3(e), the laser beam passes through L1 ($f= 25\,{\rm mm}$) and P1, then incidents to the metasurface. The output spot is collected by the second focus lens L2 and then recorded by a CCD camera. The distances between the L1 and metasurface; metasurface and L2; the L2 and the CCD camera are equal to the focal distance (25 mm). The LCP and RCP components from the metasurface split along the radial direction, pass the analyzer (P2) and focus again by L2 for the collection and are finally recorded by the CCD camera (DCC1645C, Thorlabs, USA). The intensity distribution ${I_0}( {x,y} )$ before the L1 [Fig. 3(c)] and ${I_1}( {x,y} )$ after the 4*f* system [Fig. 3(d)] are recorded separately by the CCD camera, and are consistent with the theoretical calculation results shown in Fig. 3(a) and (b) (more theoretical calculation details can be found in Note 2 of Supplementary data). The electrical field distribution can be acquired based on the equation ${E_i}( {x,y} ) \propto \sqrt {{I_i}( {x,y} )} $. The transfer function is calculated as ${\rm{H}}( {{k_x},{k_y}} ) = \frac{{{E_1}( {u,v} )}}{{{E_0}( {u,v} )}}$, where $u= x/\lambda f$and $v= y/\lambda f$. Figure 3(f) shows the transfer function result along the radial direction, in which ${k_r} = \sqrt {k_x^2 + k_y^2}$.

### 2D edge detection of amplitude object

**Figure 4.**
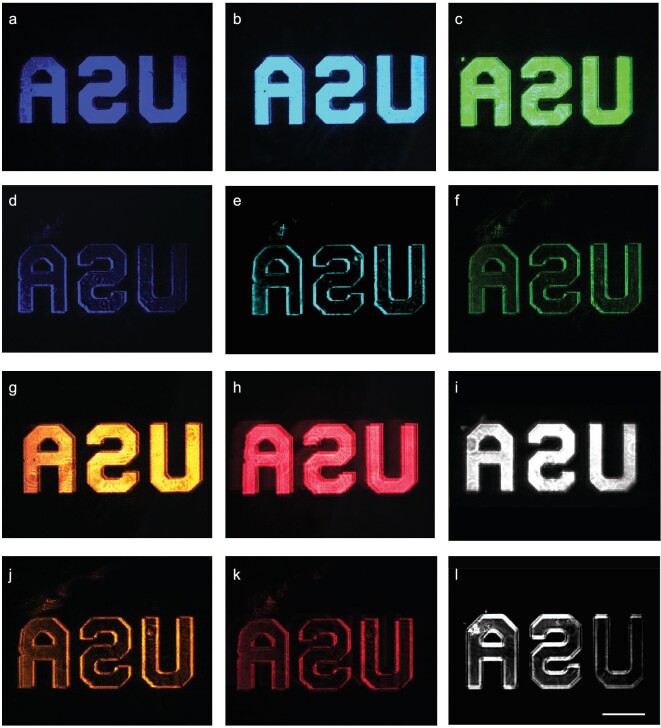
2D edge detection demonstration of intensity object (R3L3S1N Negative 1951 USAF Target). (a–c and g–i) The result is taken by removing the second polarizer. (d–f and j–l) Edge images at wavelengths of 410 nm, 490 nm, 540 nm, 590 nm, 690 nm and white light, respectively. Scale bar: 1 mm.

The experimental setup of edge detection is similar to that in Fig. 3(a), but with an object placed before the L1 at a distance of 10 cm (the focal length of L1). The light source is from a supercontinuum laser (SuperK EXW-6, NKT Photonics) coupled to a variable bandpass filter (NKT SuperK Varia High), where the incident light can be tuned from 410 to 690 nm with a 10 nm bandwidth. Figure 4(a–c) and (g–i) shows the results without the second polarizer, in which there is a small shift between two images. The theoretical prediction of the slightly separated two images shown in Fig. 4(a–c) and (g–i) can be found in Note 2 of the Supplementary data. By adding the analyzer P2, the edge information of the object is acquired, as shown in Fig. 4(d–f) and (j–l). It should be noted that the broadband property was further confirmed by a white light source. As shown in Fig. 4(i) and (l), the center wavelength of the incident light was set at 600 nm with a bandwidth of 400 nm, indicating a wavelength range from 400 nm to 800 nm (see Fig. S2 in the Supplementary data for the power density curve of the light source). As can be seen, our proposed method can efficiently block the center linear part and leave all edges. Our proposed method can work as a 2D differentiator efficiently, with the 2D edge detection covering all the visible frequencies. The broadband performance further enables spatial differentiation of color images. Here, the broadband properties of our metasurface can be explained from two points of view: the first, is that the working mechanism of our metasurface is based on birefringence rather than phase delay, in which no photonic resonance is introduced, and our phase retardation π is determined by setting the metasurface writing depth to about 50 μm; the second is attributed to our metasurface being composed of SiO_2-x_ fabricated using the pulse laser writing inside the fused silica lens. The material properties of both the SiO_2-x_ and fused silica host are weakly dispersive.

### Edge detection of a phase object

In general, the amplitude and phase of an object tend to play different roles in imaging processing, which are both important and significant. However, researchers have found that most of the important features can be preserved even if only the phase is retained [45]. As a result, much attention was given to study of the topics related to the phase object. Remarkably, in 1955, Zernike invented the phase contrast microscope in which the phase variation was converted to intensity variation by adding a phase plate [46]. It also inspired other analogy approaches for feature recognition of phase objects, such as edge detection [47–49]. However, the aforementioned edge detection methods suffer from lower contrast, limited resolution or less practicability because of complex setup requirements. Here, the edge detection image of the phase object (e.g. cells in bioimaging) with a decent quality, high-contrast and resolution is demonstrated experimentally by incorporating the metasurface with a commercial microscope.

**Figure 5.**
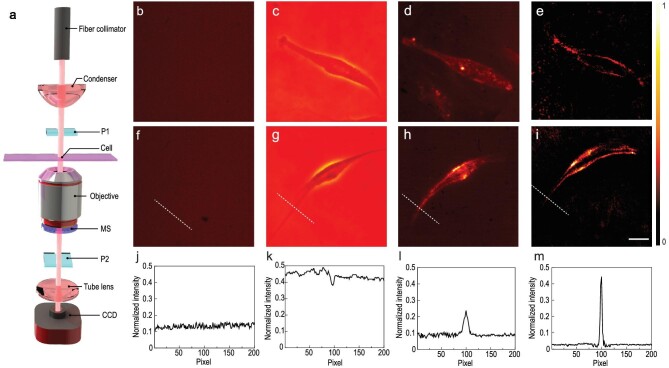
Edge detection of a phase object. (a) Measurement setup. The cell sample is placed on a glass coverslip. (b–i) Micro-scale imaging methods include bright field (b and f), phase contrast (c and g), dark field (d and h), and edge detection (e and i). The first row (b–e) is the examined human umbilical vein endothelial cell (HUVEC). The second row (f–i) is the observed human bronchial epithelial cell (HBEC). Scale bar: 100 μm. (j–m) Intensity cross-section taken along the white dashed lines in (f–i), respectively.

Figure 5(a) shows the measurement setup for edge detection of the cells. The setup is built based on a transmission Olympus microscope (IX-83) and a 532 nm, 750 mW continuous green laser (OPUS MPC 6000, Laser Quantum, England) serves as a light source. The laser is controlled by external shutters with an illumination power density under 1 W cm^−2^. The output laser is coupled to the measurement system by a fiber coupler, then it is expanded by a condenser to provide uniform illumination for the field view of the microscope. Here, we crop a small field view from the microscope and use a vibrating multimode fiber to remove the speckle to further improve the uniformity. The polarizer P1 is set before the cell to provide the LP light. The cells are imaged through an air objective (40X, 0.6NA, LUC plan FLN 40X, Olympus, Tokyo, Japan). The metasurface sample is placed at the back focal plane of the objective. The analyzer P2 is orthogonal to the P1 for elimination of the LP component, leaving out only the edge information. The images are recorded by a sCMOS camera (Orca Flash 4.0 v3 sCMOS, Hamamatsu Photonics, Japan).

We compared different imaging techniques to evaluate the proposed edge detection approach. Figure 5(b–e) and (f–i) includes several popular imaging techniques for observing human umbilical vein endothelial cells (HUVECs) and human brain endothelial cells (HBECs) grown in tissue culture vessels, respectively. Clearly, bright field images display almost no visible features because of the transparent nature of the cells (Plan N, 40X, 0.65NA, Olympus, Japan), as shown in Fig. 5(b) and (f). Figure 5(c) and (g) shows cells imaged with a phase contrast objective lens (40X, 0.65NA, Zeiss, Germany) with a phase ring in light path. Figure 5(d) and (h) shows the images of the same cells under dark-field illumination (Objective lens, LD EC Epiplan-Neofluar, 50X, 0.55NA, Zeiss, Germany), which contain only the scattered light from the cell. Figure 5(e) and (i) presents the edge detection results of our proposed approach. As shown in Fig. 5(j–m), the intensity cross-section of Fig. 5(f–i) is provided. Compared with dark-field and phase contrast techniques, our method exhibits clear and strong signals at the cell edges, indicating exceptionally high sensitivity and precision to detect the transparent biological specimens.

## DISCUSSION AND CONCLUSION

We present the first broadband 2D spatial differentiator based on a dielectric metasurface, which enables high-contrast edge imaging across the whole visible spectrum. Furthermore, we extend the detection from intensity object to phase object for both theory and experiment. Our design also has other advantages, such as operation in transmission mode for the whole system, which is more compatible with a standard image processing system. Also, the proposed edge detection is not limited to one single wavelength because of employment of dielectric material rather than relying on resonance phenomena. Its multi-frequencies enable differentiation of color images. In conclusion, we have shown that 2D spatial differentiation enabled edge detection of intensity and phase objects can be implemented using

a designed dielectric metasurface, and such a design may have broad applications in the field of analog image processing.

## METHODS

### Birefringence measurement

The birefringence of the sample was conducted with a quantitative birefringence measurement system (ABRIO, CRI Inc.) integrated into an optical microscope microscopy (BX51, Olympus Inc.). A circularly polarized light from a halogen lamp, passing through a bandpass filter (center wavelength 633 nm and bandwidth of 30 nm) was employed to illuminate the sample. The sample was measured with an analyzer in the plane perpendicular to the laser propagation direction. The birefringence, including the phase retardance and orientation of the slow axis, were extracted [50].

### Cell growth

HBEC-5i cells (ATCC^®^ CRL-3245™) from human brain and human umbilical vein endothelial cells (HUVECs, Lonza C2519A) were separately cultured in EGM-2 endothelial cell growth medium (Lonza CC3162). Cells used for this study were all below passage 5. To prepare for microscopy, cells were placed on two Millicell EZ 8-well glass slides (Millipore Sigma PEZGS0816) at 50 000 cells/mL and cultured in EGM-2 medium overnight at 37°C in a humidified incubator to allow for cell attachment. On the next day, cells were rinsed with phosphate buffered saline (PBS) once and fixed in 4% paraformaldehyde (PFA) solution (Fisher Scientific AAJ19943K2) for 10 minutes at room temperature. Cells were then rinsed three times with PBS to remove all PFA residual. After fixation, the wells and slide holders of EZ slides were removed, leaving only the slides with fixed cells. The slides were mounted with VECTASHIELD^®^ antifade mounting media (Vector Laboratory H1000) and covered with a cover glass. The mounted slides were dried in a chemical hood for 20 minutes and stored at 4°C until imaging.

## Supplementary Material

nwaa176_Supplemental_File
