# Supplementary material for: Two-dimensional optical spatial differentiation and high-contrast imaging
Source: Natl Sci Rev. 2020 Aug 6;8(6):nwaa176. doi: 10.1093/nsr/nwaa176 (PMC8288167; doi:10.1093/nsr/nwaa176)
Supplement: nwaa176_Supplemental_File [file nwaa176_supplemental_file.pdf]

Supplementary Information for

Two-dimensional optical spatial differentiation and high-contrast imaging

Junxiao Zhou, Haoliang Qian, Junxiang Zhao, Min Tang, Qianyi Wu, Ming Lei, Hailu Luo,

Shuangchun Wen, Shaochen Chen and Zhaowei Liu*

Corresponding authors:

**Hailu Luo* hailuluo@hnu.edu.cn *and *Zhaowei Liu* zhaowei@ucsd.edu

Contents:

Note 1. Theory of the metasurface enabled LCP and RCP splitting

Note 2. Theoretical calculation without and with the spatial differentiator.

Figures S1 and S2

Note 1. Theory of the metasurface enabled LCP and RCP splitting

The laser writing pattern inside of the metasurface can create an artificial homogeneous phase retardation π. The local optical and slow and fast axes at each point are oriented parallel and perpendicular to the subwavelength structures, respectively. The orientation of the optical slow axis follows the relation of $\varphi(x,y) = \frac{\pi\sqrt{x^2+y^2}}{\Lambda}$, where $\Lambda = 1000$ μm is the period of waveplate. Here, we employ the wave-optics methods to analyze the spin-orbit interaction induced by the wave plate, where its Jones matrix T can be given as :

$$T = \begin{bmatrix} \cos 2\varphi & \sin 2\varphi \\ \sin 2\varphi & -\cos\ 2\varphi \end{bmatrix}$$

Suppose one LCP ($|\mathrm{L}\rangle$) or RCP ($|\mathrm{R}\rangle$) normally impinges onto the waveplate (LCP and RCP refer to left and right-handed circular polarization), the output states could be calculated as: $|E_{out}\rangle = T(x,y)(|\mathrm{L}\rangle) = \exp(i2\varphi)\,|R\rangle$ and $|E_{out}\rangle = T(x,y)(|\mathrm{R}\rangle) = \exp(-i2\varphi)\,|L\rangle$, respectively. Here, $|L\rangle = (1,i)^T/\sqrt{2}$ and $|R\rangle = (1,-i)^T/\sqrt{2}$. As one can see, the handedness of the incident photons is inverted, and an additional space-variant phase, i.e. PB phase (Pancharatnam-Berry phase), $\Phi_{PB} = \pm 2\varphi(x,y)$ is induced. This phase depends on the location of the orientation of the optical axes and its gradient will steer different handedness photons to opposite directions:$\Delta\mathrm{k} = -\nabla\Phi_{PB}$. Consequently, the wavefront is manipulated and the wave centroid along the radial direction owns a spatial shift $\Delta = \frac{\Delta \mathrm{k}}{\mathrm{k}_0}\mathrm{z} = \pm\frac{\lambda}{\Lambda}\mathrm{z}$, where $\mathrm{k}_0 = \frac{2\pi}{\lambda}$, λ is the wavelength and z is the propagation distance.

Note 2. Theoretical calculation with and without the spatial differentiator.

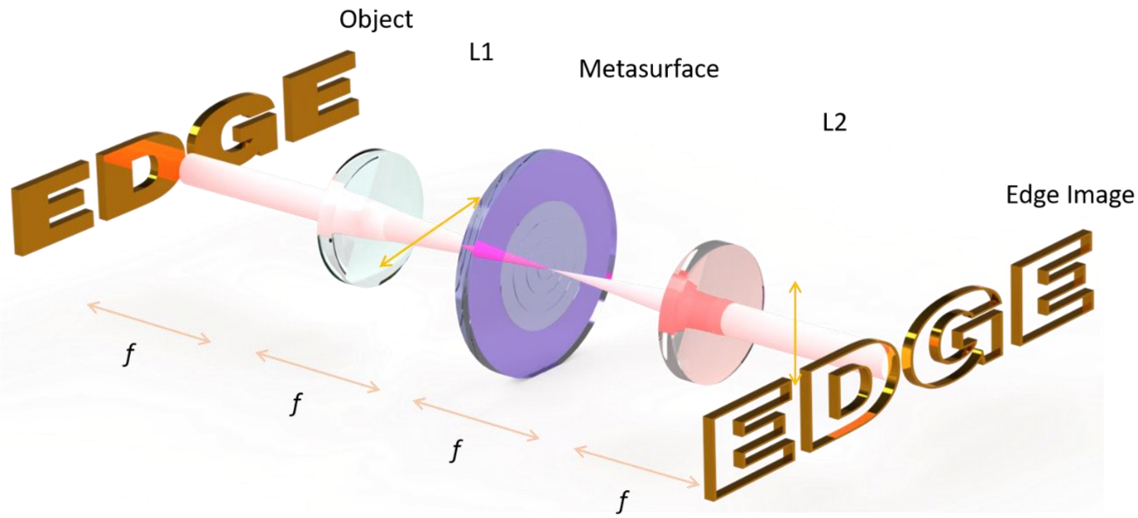

Figure S1. The work concept of the two-dimensional edge detection. P, plane; L, lens. A Pairs of yellow double-headed arrows indicate orthogonal polarization states before and after the metasurface.

As shown in Figure S1, the first lens (L_1) computes the Fourier transform of the electric field at the object plane. The metasurface is located at the Fourier plane of the 4f system, where the Fourier spectrum is formed. The second lens (L_2) takes the inverse Fourier transform of the spectrum modified by the metasurface and creates the output edge information at its back focal plane. To illustrate the calculation process, assume the incident light is x-polarized, the input electric field of the object is defined as $E_0(r_{1,}\theta_1)\begin{pmatrix}1\\0\end{pmatrix}$. After propagating through L_1, the electric field $E_1(r_{1,}\theta_1)$ right in front of the metasurface is given as:$E_1(r_{2,}\theta_2) = \mathcal{F}[E_0(r_{1,}\theta_1)]$, where $\mathcal{F}$ is the Fourier transform operator. Considering the spatial differentiation function of the metasurface, the electric field right behind the metasurface is $E_2(r_{2,}\theta_2) = E_1(r_{2,}\theta_2)\ \left[\exp\left(i * \frac{2\pi}{\Lambda} * r\right)\begin{pmatrix}1\\-i\end{pmatrix} + \exp\left(-i * \frac{2\pi}{\Lambda} * r\right)\begin{pmatrix}1\\i\end{pmatrix}\right]$. Here, Λ is the period of the metasurface; these two terms, $\exp\left(i * \frac{2\pi}{\Lambda} * r\right)$ and $\exp\left(-i * \frac{2\pi}{\Lambda} * r\right)$ are the PB phase achieved by metasurface for LCP and RCP components; for terms, $\begin{pmatrix}1\\i\end{pmatrix}$ and $\begin{pmatrix}1\\-i\end{pmatrix}$, these are Jones vectors for LCP and RCP components. To achieve the edge information, two orthogonal polarizers are employed in our system. Therefore, the electric field $E_2(r_{2,}\theta_2)$ could be further given as $E_2(r_{2,}\theta_2) = E_1(r_{2,}\theta_2)\ \left[\exp\left(i * \frac{2\pi}{\Lambda} * r\right) - \exp\left(-i * \frac{2\pi}{\Lambda} * r\right)\right] = E_1(r_{2,}\theta_2)\sin\left(\frac{2\pi}{\Lambda} * r\right)$. After the propagation through L_2, the electric field at the image plane is derived from $E_3(r_{3,}\theta_3) = \mathcal{F}[E_2(r_{2,}\theta_2)]$. The light intensity of the output image is given as

$I_{out} = \left|E_3(r_{2,}\theta_2)\right|^2$, which is shown in Figure 3b. To obtain the intensity distribution without the spatial differentiator, the modification of the Fourier spectrum from the metasurface was not introduced, and the results are shown in Figure 3a.

It should be noted that for our imaging system, if two orthogonal polarizers are changed to co-polarized state or the second polarizer is removed, the mentioned electric field $E_2(r_{2,}\theta_2)$ behind the metasurface will be modified as $E_2(r_{2,}\theta_2) = E_1(r_{2,}\theta_2)\ \left[\exp\left(i * \frac{2\pi}{\Lambda} * r\right) + \exp\left(-i * \frac{2\pi}{\Lambda} * r\right)\right] = E_1(r_{2,}\theta_2)\cos\left(\frac{2\pi}{\Lambda} * r\right)$. After propagation through $L_{2,}$ the electric field at the image plane is written as $E_3(r_{3,}\theta_3) = \mathcal{F}[E_2(r_{2,}\theta_2)]$. The finial intensity distribution is given as $I_{out} = \left|E_3(r_{2,}\theta_2)\right|^2$. We can expect that the two separated images will be obtained instead of edge images, as shown in Figures 4a-c and g-i in the manuscript.

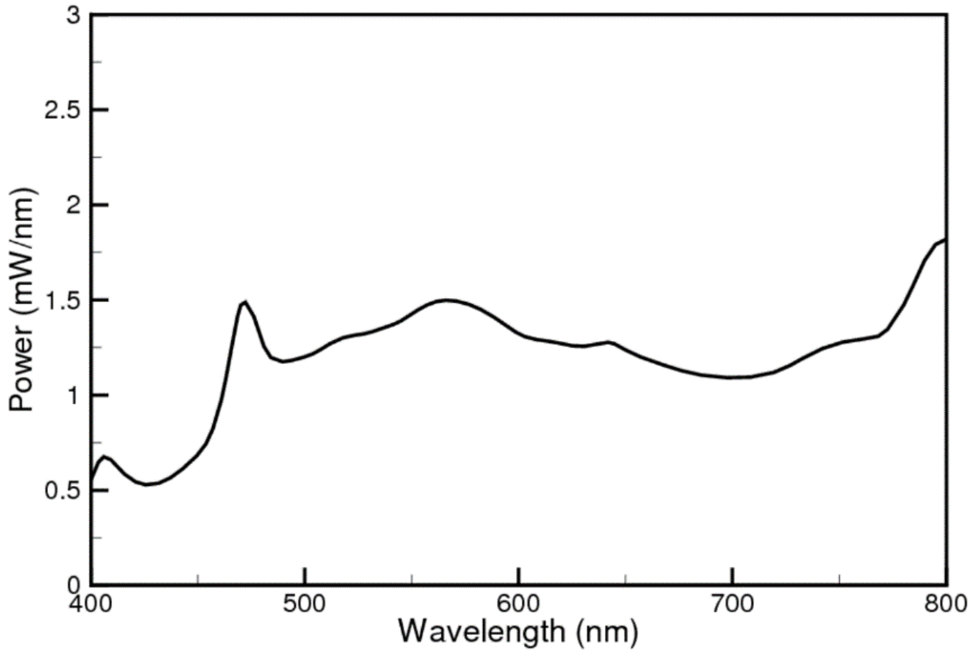

Figure S2. The power density curve of the working wavelength range.
